# Supplementary material for: Three body photodissociation of the water molecule and its implications for prebiotic oxygen production
Source: Nat Commun. 2021 Apr 30;12:2476. doi: 10.1038/s41467-021-22824-7 (PMC8087761; doi:10.1038/s41467-021-22824-7)
Supplement: Supplementary file 1 — Supplementary Information [file 41467_2021_22824_MOESM1_ESM.pdf]

Supplementary Information

Three Body Photodissociation of the Water Molecule and its Implications for Prebiotic Oxygen Production

Chang et al

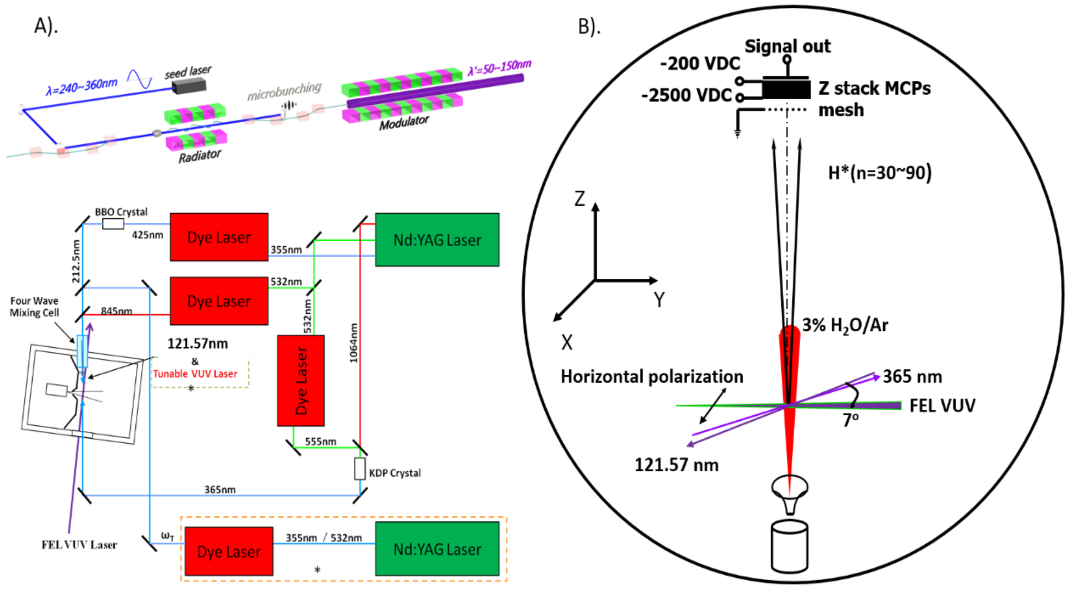

Supplementary Figure 1. Schematic of the DCLS beam line (A) and the VUV FEL-HRTOF photodissociation experimental setup (B). The VUV-FEL facility at the DCLS generates the radiation with the wavelength continuously tuning between 50-150 nm. This radiation crosses perpendicularly with the H_2O molecular beam, which is generated by expanding a 3% mixing of H_2O and Ar at a stagnation pressure of 600 Torr through a pulsed nozzle. About 5 ns after the VUV-FEL photodissociating H_2O, the H-atom products are excited to a high Rydberg state via two-step resonant excitation by absorbing the photons of 121.57 nm and 365 nm.

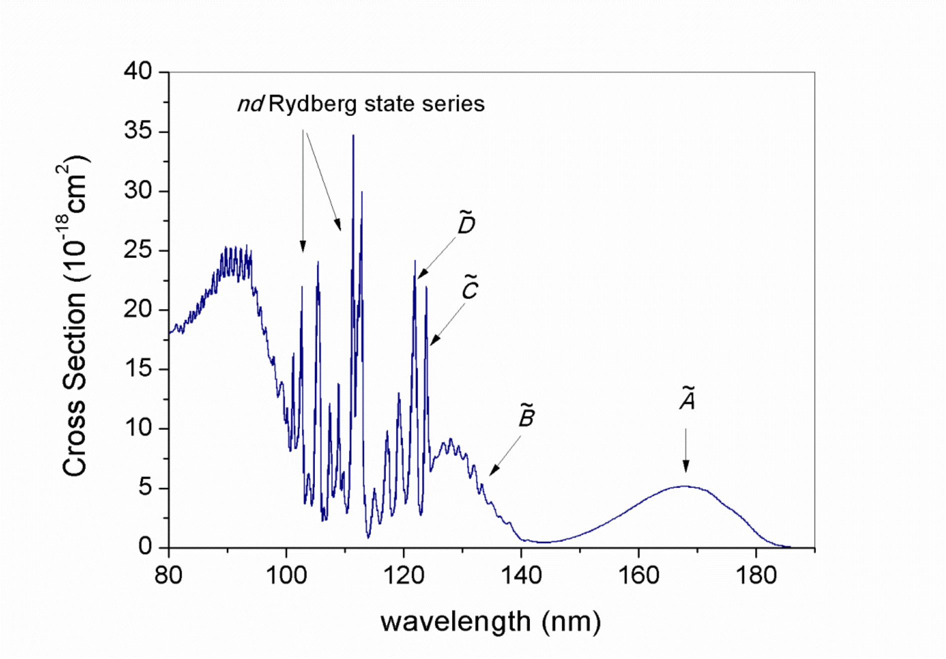

Supplementary Figure 2. Absorption spectrum of H_2O vapor at room temperature[1].

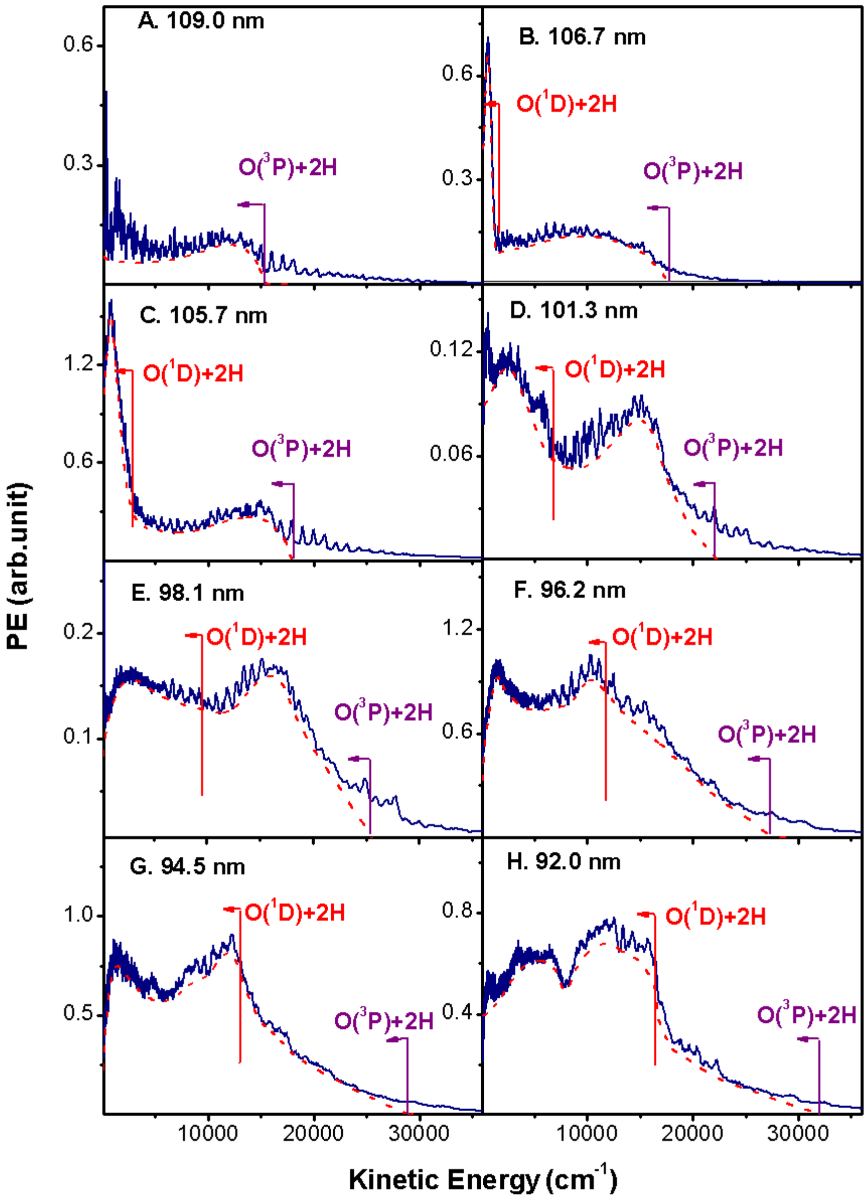

Supplementary Figure 3. Experimental TKER spectra (blue solid curve) derived from H atom TOF measurements following photodissociation of H_2O at λ = 109.0, 106.7, 105.7, 101.3, 98.1, 96.2, 94.5 and 92.0 nm with the detection axis aligned 54.7° (magic angle) to the polarization direction of the VUV FEL beam, along with the simulated profiles (red dashed curve) for the TBD leading to $O(^1D)$ + 2H and $O(^3P)$ + 2H products. The uncertainty in the fraction of the total signal attributed to the TBD is estimated to be no more than ±10% in the branching fractions reported in Table 1.

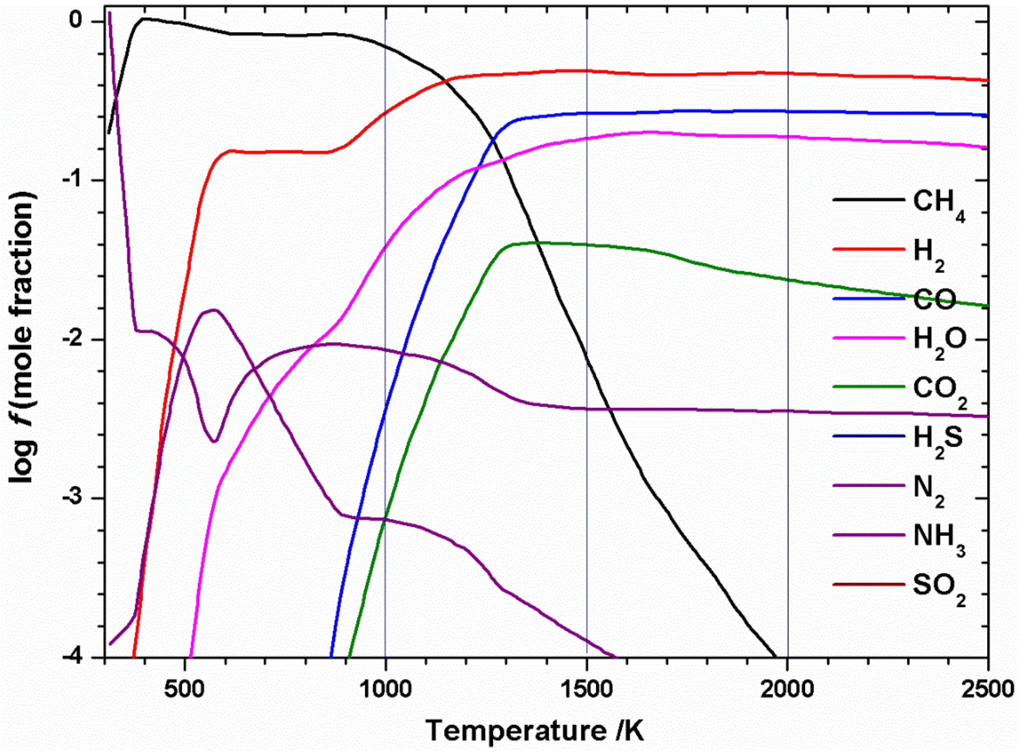

Supplementary Figure 4. Gas composition in equilibrium with ordinary H-type chondrites at a pressure p = 100 bar. Ordinary chondrites are often regarded as indicative of the bulk material of the Earth. The gases are reduced and methane is strongly favored at p = 100 bar[2].

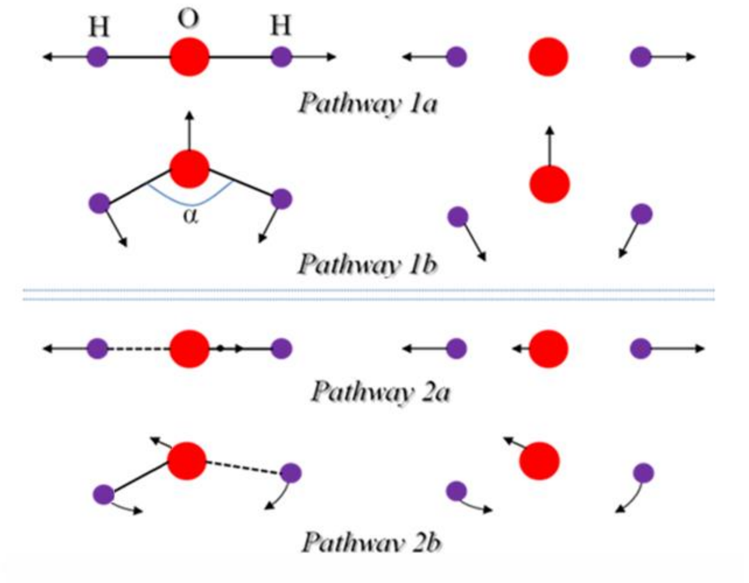

Supplementary Figure 5. Schematic of possible TBD pathways of H_2O. The top panel depicts the simultaneous processes, in which three fragments (an O atom and two H atoms) are released simultaneously. The lower panel shows the sequential processes, wherein the initial dissociation yields a 'hot' (*i.e.* highly vibrationally and/or rotationally excited) intermediate OH species.

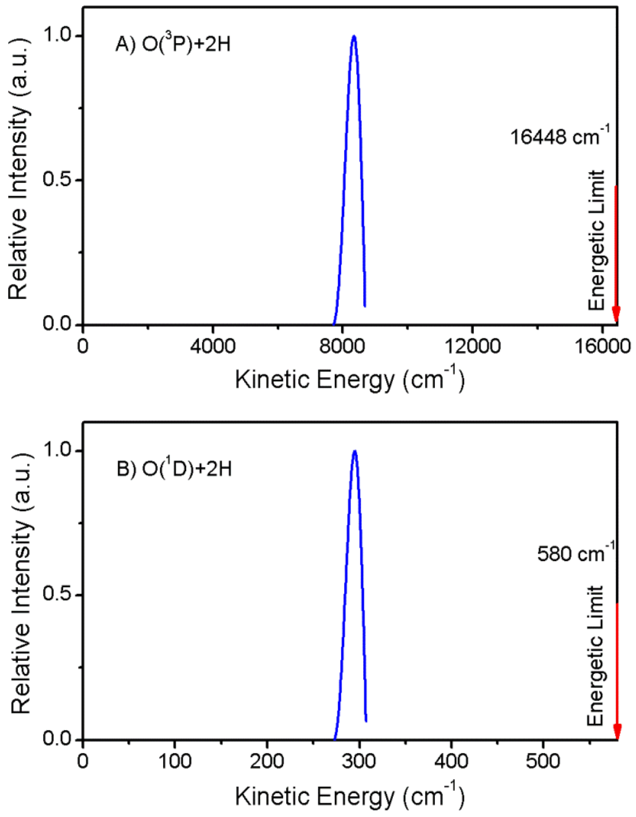

Supplementary Figure 6. Simulated kinetic energy spectra of the O(^{1}D) + 2H and O(^{3}P) + 2H products arising from the simultaneous dissociation of H_2O molecules with an equilibrium bond angle of 109° (appropriate for the *nd* Rydberg states[3]). Calculations for other initial bond angles yield very similar kinetic energy distributions.

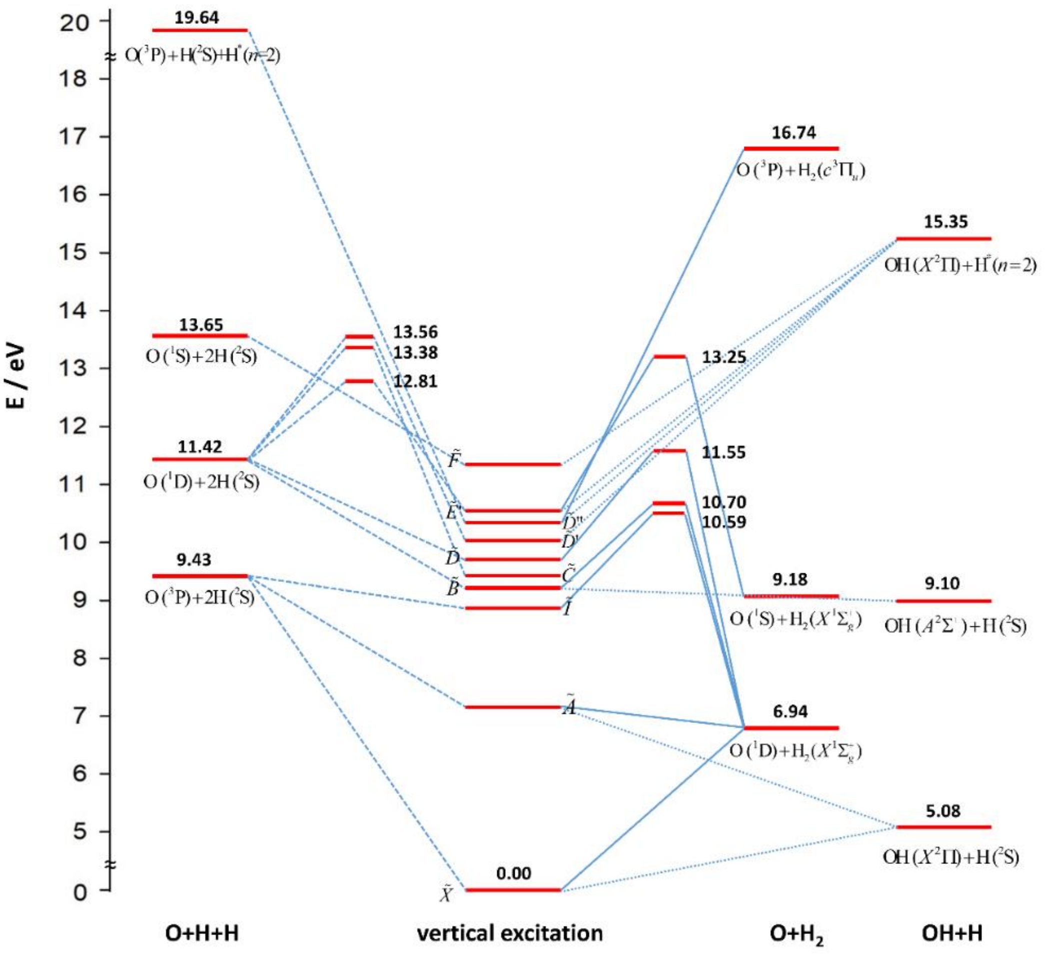

Supplementary Figure 7. Energy level diagram for various binary and TBD channels of H_2O. The quoted excited state energies are for the respective zero-point (*i.e.* v=0) levels, defined relative to the $\tilde{X}$, v=0 level.

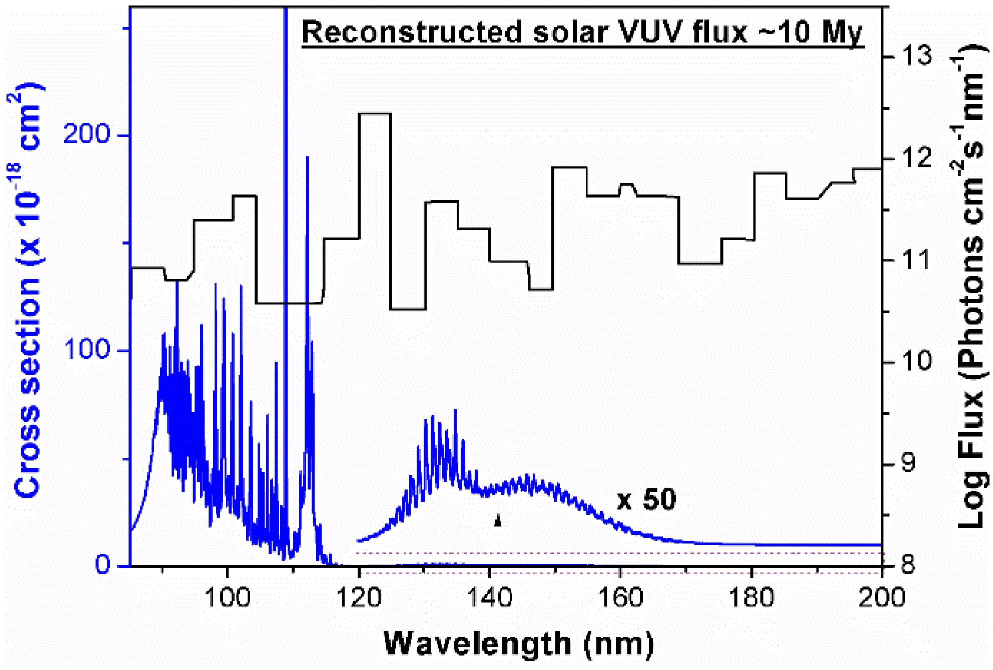

Supplementary Figure 8. Plot showing the wavelength dependences of the reconstructed VUV solar flux (90-200 nm) at ~10My, the total absorption (σ_{tot}) cross-sections of CO_2, and assuming the quantum yield for forming oxygen atom photoproducts being unity. We assume the total absorption cross-section is the same as the photodissociation cross section for CO_2, due to the predissociation rate of CO_2 is sufficiently fast that the fluorescence quantum yield must be negligible.

Supplementary Note 1

Experimental methods. The experiments employ a newly constructed apparatus for molecular photochemistry, which is centered on the vacuum ultraviolet free electron laser (VUV-FEL) beam line at the Dalian Coherent Light Source (DCLS) [4]. Briefly, the VUV-FEL facility runs in the high gain harmonic generation (HGHG) mode, in which the seed laser is injected to interact with the electron beam in the modulator (Supplementary Figure 1). The seeding pulse, in the wavelength range (λ_{seed}) 240-360 nm, can be generated from a picosecond Ti:sapphire laser pulse. The electron beam is generated from a photocathode RF gun, and accelerated to the beam energy of ~300 MeV by 7 S-band accelerator structures, with a bunch charge of 500 pC. The micro-bunched beam is then sent through the radiator, which is tuned to the 2nd/3rd/4th harmonic of the seed wavelength, and coherent FEL radiation with wavelength $\lambda_{seed}/2$, $\lambda_{seed}/3$ or $\lambda_{seed}/4$ is emitted. Optimization of the linear accelerator yields a high quality electron beam with emittance of ~1.5 mm·mrad, energy spread of ~1‰, and pulse duration of ~1.5 ps. In this work, the VUV-FEL operates at 10 Hz, and the maximum pulse energy is >100 μJ/pulse. The output wavelength is continuously tunable in the range 50-150 nm and the typical spectral bandwidth of the VUV-FEL output is 30~50 cm^{-1}.

The high-n H atom Rydberg tagging time-of-flight (HRTOF) technique used in this work was pioneered by Welge and coworkers[5]. The key point of this technique is the 1+1′ (VUV+UV) excitation of the H atom. The first step involves VUV laser excitation of the H atom from its n=1 ground state to the n=2 state by absorbing one λ = 121.57 nm photon. In the second step, the H (n=2) atom is excited with a UV (λ ~365 nm) photon to a high-n (n=30-80) Rydberg state. The 121.57 nm photon is generated by four wave difference frequency mixing using two 212.5 nm photons and one 845 nm photon in a cell containing a Kr/Ar gas mixture (1:3 mixing ratio). The 212.5 nm laser light is produced by doubling the output of a tunable dye laser operating at ~425 nm, pumped by the third harmonic output of a Nd:YAG laser, while the 845 nm laser light is the direct output of another dye laser pumped by part of the second harmonic output from the same Nd:YAG laser. The 365 nm laser light is generated by doubling the output of

a tunable dye laser operating at ~730 nm, pumped by another part of the second harmonic output of the Nd:YAG laser.

Charged species formed in the interaction region are extracted from the TOF axis by a small electric field (~20V/cm) placed across this region. Rydberg tagged neutral H atoms fly a known distance (d ≈280 mm) from the interaction region to a rotatable microchannel plate (MCP) Z-stack detector located close behind a grounded fine metal grid. After passing through the grid, the Rydberg atoms are immediately field-ionized by the electric field (~2000V/cm) applied between the grid and the front plate of the Z-stack MCP detector. The signal detected by the MCP is amplified by a fast pre-amplifier and counted by a multichannel scaler. A molecular beam of H_2O is generated by expanding a mixture of H_2O and Ar at a stagnation pressure of 600-900 Torr through a 0.5 mm diameter pulsed nozzle (General Valve). The FEL output is orthogonal to the molecular beam axis, and polarized in the horizontal direction. TOF spectra along axes parallel and perpendicular to this polarization axis are thus measured by rotating the MCP detector. The 121.57 nm detection laser pulse causes some H_2O photodissociation. The TOF spectrum of these probe laser induced H atoms is obtained by turning the VUV-FEL beam on and off, and subtracted accordingly.

Supplementary Note 2

The VUV absorption spectrum of H_2O. Supplementary Figure 2 shows the absorption spectrum of H_2O at room temperature. The H_2O molecule in its ground ($\tilde{X}$ $^1A_1$) state has C_{2v} symmetry and electronic configuration: $(1a_1)^2(2a_1)^2(3a_1)^2(1b_1)^2$. It displays richly structured absorption at VUV wavelengths λ <190 nm as a result of the excitation of one electron from the $1b_1$ or $3a_1$ orbital to a Rydberg orbital. The absorption spectrum in the ~190-120 nm region consists of two broad, well-separated bands with maxima at ~167 nm and ~128 nm, respectively[6]. These bands are assigned to, respectively, the $\tilde{A}^1B_1 \leftarrow \tilde{X}^1A_1$ ($3sa_1 \leftarrow 1b_1$) and $\tilde{B}^1A_1 \leftarrow \tilde{X}^1A_1$ ($3sa_1 \leftarrow 3a_1$) transitions. The first intense, sharp bands peaking at ~124 nm and ~122 nm are attributed to, respectively, the $\tilde{C}^1B_1 \leftarrow \tilde{X}^1A_1$ ($3pa_1 \leftarrow 1b_1$) and $\tilde{D}^1A_1 \leftarrow \tilde{X}^1A_1$ ($3pb_1 \leftarrow 1b_1$) Rydberg transitions and most of the sharp features in the ~118-92 nm region are attributed to $nd \leftarrow 1b_1$ ($n \geq 3$) excitations[7].

Supplementary Note 3

Experimental results at eight more VUV wavelengths. Supplementary Figure 3 shows TKER spectra derived from H atom TOF spectra following photodissociation of H_2O at λ = 109.0, 106.7, 105.7, 101.3, 98.1, 96.2, 94.5 and 92.0 nm, respectively, with the detection axis aligned at 54.7° (magic angle) to the polarization direction of the VUV FEL beam. The simulated profiles for the TBD (O(^{1}D) + 2H and O(^{3}P) + 2H fragments) are shown by the red dashed curves. The divided profiles for each TBD channel are a little arbitrary due to severe overlap between the two three-body channels at shorter wavelengths, thus are not shown here. The derived branching ratios for the binary and TBD channels are shown in Table 1. It is noted that the branching ratio for the binary and TBD at 105.7 nm shows a deviation from the general trend, *i.e.*, the binary channel from 105.7 nm photolysis is relatively larger than that from its neighboring wavelengths. Such dynamical source is not immediately clear, since the potential energy surfaces of high Rydberg states of water are lacking. As reported by Fillion et al.,[7] the intense features of the absorption spectrum (98-114 nm), are dominated by the $nd\leftarrow 1b_1$ transition series. At 105.7 nm, the water molecule was excited to 4d Rydberg state with $^1B_2$ symmetry. A bent-linear interaction between this $^1B_2$ state and the $^1B_2$ $3pb_2$ state (the state of which has a quasilinear geometry and arises from the excitation of the $3a_1$ orbit) may occur. Thus, the water molecule can undergo a fast conversion from the initial excited Rydberg state to the $^1B_2$ state via an avoid crossing between them[3], and then predissociate to the dissociative $^1A_2$ $3pb_2$ state due to Renner-Teller coupling between the $^1B_2$ and $^1A_2$ states. The molecules on the $^1A_2$ state can further couple to the $\tilde{A}$ state yielding H+OH(X) products. While at other photolysis wavelengths, the water molecules mainly undergo a fast dissociation on the $\tilde{B}^1A_1$ state surface after multi-step internal conversions from the initial excited Rydberg state to the $\tilde{B}$ state.

Supplementary Note 4

The relative abundances of different gases in the early atmosphere of the Earth. The origin of Earth's atmosphere is a profound question. The conventional wisdom is that the earliest atmosphere of the Earth arose from the outgassing of volatiles from minerals during and/or after planetary accretion. Recent studies have reported outgassing of ordinary chondritic material and the implications of such measurements for the composition of Earth's early atmosphere[2, 8]. Supplementary Figure 4 shows gas compositions obtained by heating ordinary chondritic material to the solidus temperature (T ~1225 K)[9]. The abundance of gas phase H_2O is found to be >10 times that of CO_2 at T ~1000 K, which is a plausible surface temperature for the nascent Earth. The formation of the Earth's core due to gravitational sinking may have released tremendous amounts of heat, raising the Earth's surface temperature. Norman *et al.*[2] suggested that the surface temperature of the nascent Earth may have reached ~2300 K prior to gradual radiative cooling, and we note that the photochemical model introduced by Yung *et al.*[10] assumed an H_2O abundance of ~50 times greater than that of CO_2. In the estimation of the relative oxygen yields from H_2O and CO_2 in Earth's primitive atmosphere, we assumed that H_2O is 10 times more abundant than CO_2.

Supplementary Note 5

Possible TBD pathways for H_2O. Oxygen atom production from H_2O molecules could, in principle, occurs via a simultaneous or a sequential TBD process (Supplementary Figure 5). In the former case, *pathway 1* in Fig. S4 ($H_2O \rightarrow O + H + H$, see below), the two O–H bonds break simultaneously and the two H atom fragments should be formed with equal kinetic energies. In contrast, in a sequential dissociation, *pathway 2* in Fig. S5 (*i.e.*, $H_2O \rightarrow H + OH^* \rightarrow O + H + H$), the H_2O molecule first splits into an H atom and a rovibrationally excited OH* radical and the latter then dissociates to yield an O atom and a second H atom. In either case, we can consider limiting models involving either a linear or nonlinear predissociative transition state of H_2O, as illustrated in Supplementary Figure 5.

Focussing first on the *pathway 1* case, the kinetic energies of any H atoms produced via such a simultaneous TBD process can be derived using momentum and energy conservation arguments[11], *i.e.*

$$E_{KE} = \frac{E_a}{2\times\left(1+2\times\left(\frac{m_H}{m_O}\right)\cos^2\left(\frac{\theta}{2}\right)\right)} \qquad \text{(S1)}$$

where m_H and m_O are the respective atomic masses, E_a is the available energy (*i.e.* $E_{h\nu} + E_{int}(H_2O) - E_{th}$, where E_{th} is the threshold energy for the TBD process of interest) and θ is the molecular inter-bond angle. As Supplementary Figure 6 shows, the TKER spectra predicted for this scenario using Eq. S1 consist of narrow peaks centred at kinetic energy values close to one half of E_a (*i.e.*, at ~290 cm^{-1} and at ~8224 cm^{-1} for the $O(^1D) + 2H$ and $O(^3P) + 2H$ channels, respectively). The big mass difference between the H and O atoms ensures that the two H atoms each recoil with almost (or, in the limit that $\theta = 180°$ (*pathway 1a*) exactly) half of the total available energy. This is clearly not the case in the present experiment (see Figure 1 in the main text), implying that sequential three-body dissociations (*pathway 2* in Supplementary Figure 5) are the dominant source of the observed atomic products.

Supplementary Note 6

Electronic Structure Considerations. To shed further insights into the photodissociation mechanisms of H_2O molecules from higher excited states, we have calculated ten adiabatic potential energy surfaces (PESs) of H_2O (five $^1A'$ electronic states $\tilde{X}$, $\tilde{B}$, $\tilde{D}$, $\tilde{E}'$, and $\tilde{F}$, and five $^1A''$ electronic states $\tilde{A}$, $\tilde{I}$, $\tilde{C}$, $\tilde{D}'$, and $\tilde{D}''$ in the C_s point group) using the internally contracted multi-reference configuration interaction method with the Davidson correction (icMRCI+Q) and a large basis set[12-14].

Supplementary Figure 7 shows the energy level diagram correlating the calculated vertical excitation energies of these excited states of H_2O and the various binary and three-body dissociation limits. The $\tilde{X}$, $\tilde{A}$, and $\tilde{I}$ states correlate to the O(^{3}P) + 2H asymptote (where $\tilde{I}$ represents $^1A_2(3pb_2\leftarrow 1b_1)$ state), while the $\tilde{B}$, $\tilde{C}$, $\tilde{D}$, $\tilde{D}'$, and $\tilde{E}'$ states are predicted to correlate with the O(^{1}D) + 2H product asymptote. It is noted that the $\tilde{C}$, $\tilde{D}'$, and $\tilde{E}'$ states have large barriers along the O(^{1}D) + 2H dissociation pathway.

Previous studies[3, 15-17] have suggested that the *nd* Rydberg states excited in the present work may undergo fast internal conversion to the $\tilde{D}$ state PES, and further to lower lying electronic states, *i.e.*, the $\tilde{B}$ and $\tilde{X}$ states. In addition to the documented avoided crossing between the $\tilde{D}$ and $\tilde{B}$ states at a bond angle of about 100°[18], the present calculations find a conical intersection seam between the adiabatic PESs for the $\tilde{D}$ and $\tilde{B}$ states at C_{2v} geometries and O–H bond lengths in the range 0.95-1.6 Å, with a minimum energy of ~11.04 eV at R_{OH} = 1.12 Å and θ_{HOH} =78 °, providing clear theoretical evidence of fast conversion from $\tilde{D}$ to $\tilde{B}$. Thus, H_2O molecules on the $\tilde{D}$ state PES could dissociate in several possible ways: (1) Direct dissociation on the $\tilde{D}$ state PES to yield the three-body dissociation products O(^{1}D) + 2H. This dissociation pathway has no barrier, which may explain the relatively large recoil anisotropy of the observed O(^{1}D) + 2H products. (2) Fast non-adiabatic coupling to the $\tilde{B}$ state and direct dissociation on the $\tilde{B}$ state PES to yield the binary fragments OH(A) + H. If the

OH(A) products acquire sufficient internal energy they can dissociate further to produce an O(^{1}D) atom and a second H atom. This process should be expected to have a relatively smaller angular anisotropy. (3) Fast internal conversion to the $\tilde{B}$ state, followed by further non-adiabatic coupling to the $\tilde{X}$ state via either of the two documented conical intersections between the $\tilde{B}$ and $\tilde{X}$ state PESs, leading to both binary (OH(X) + H) and three-body (O(^{3}P) + 2H) dissociations. Earlier trajectory calculations by Dixon[19] suggested that almost all the trajectories leading of the O(^{3}P) + 2H products couple to the ground state PES at near linear O–H–H geometries, and that the dissociation is sequential, via an H + O–H intermediate step, in good accord with the present experimental findings. However, accurate dynamics calculations for three-body dissociations from highly excited Rydberg states are not possible at this time.

Supplementary Note 7

Oxygen production rate for CO_2 VUV photochemistry. We have drawn the wavelength dependences of the solar photon flux in the early period (10 My=1×10^7 year)[20], the total photoabsorption cross sections of the parent CO_2 molecule in the VUV region (90-200 nm)[21], and assuming the production yields of O-atom being unity, as shown in Supplementary Figure 8. The photodissociation rate constant for O production: J_{CO2}=~1.8×10^{-4} s^{-1}. Then this gives the O production rate: $N_{CO2}(O)$= $J_{CO2}\times[CO_2]=1.8\times10^{-4}[CO_2]$, where $[CO_2]$ is the density of CO_2.

Supplementary References:

1. Lee, L. C. & Suto, M. Quantitative photoabsorption and fluorescence study of H_2O and D_2O at 50-190 nm. *Chem. Phys.* **110**, 161-169 (1986).
2. Schaefer, L. & Fegley, B. Chemistry of atmospheres formed during accretion of the Earth and other terrestrial planets. *Icarus,* **208,** 438-448 (2010).
3. Hirst, D. M. & Child, M. S. Ab initio bending potential energy curves for Rydberg states of H_2O. *Mol. Phys.* **77**, 463-476 (1992).
4. H. L. Wang, et al., Photodissociation dynamics of H_2O at 111.5 nm by a vacuum ultraviolet free electron laser. *J. Chem. Phys.* **148**,124301 (2018).
5. Schnieder, L., Meier, W., Welge, K. H., Ashfold, M. N. R. & Western, C. M. Photodissociation dynamics of H_2S at 121.6 nm and a determination of the potential energy function of $SH(A^2\Sigma^+)$. *J. Chem. Phys.* **92**, 7027-7037 (1990).
6. Mota, R. et al., Water VUV electronic state spectroscopy by synchrotron radiation. *Chem. Phys. Lett.* **416**, 152-159 (2005).
7. Fillion, J. H. et al., High resolution photoabsorption and photofragment fluorescence spectroscopy of water between 10.9 and 12 eV. *J. Chem. Phys.* **120**, 6531-6541 (2004).
8. Schaefer, L. & Fegley, B. Outgassing of ordinary chondritic material and some of

its implications for the chemistry of asteroids, planets, and satellites. *Icarus* **186**, 462-483 (2007).

9. Schaefer, L. & Fegley, B. Volatile element chemistry during metamorphism of ordinary chondritic material and some of its implications for the composition of asteroids. *Icarus* **205**, 483-496 (2010).
10. Yung, Y. L. & Demore, W. B. *Photochemistry of Planetary Atmosphere* (Oxford Univ. Press, New York, 1999).
11. Maul, C. & Gericke, K. H. Photo induced three body decay. *Int. Rev. Phys. Chem.* **16**, 1-79 (1997).
12. Werner, H. J. & Knowles, P. J. An efficient internally contracted multiconfiguration reference configuration interaction method. *J. Chem. Phys.* **89**, 5803-5814 (1988).
13. Knowles, P. J. & Werner, H. J. An efficient method for the evaluation of coupling coefficients In configuration interaction calculations. *Chem. Phys. Lett.* **145**, 514-522 (1988).
14. Davidson, E. R. & Silver, D. W. Size consistency in dilute helium gas electronic structure. *Chem. Phys. Lett.* **52**, 403-406 (1977).
15. Chang, Y. et al., Hydroxyl super rotors from vacuum ultraviolet photodissociation of water. *Nat. Commun.* **10**, 1250 (2019).
16. Theodorakopoulos, G. et al., Bending potentials for H_2O in the ground and the first six singlet excited states. *Chem. Phy.s Lett.* **105**, 253-257 (1984).
17. Petsalakis, I. D., Theodorakopoulos, G. & Child, M. S. Ab initio multichannel quantum defects for the $^1A_1$ Rydberg states of H_2O. *J. Phys. B: At. Mol. Opt.* **28**, 5179-5192 (1995).
18. van Harrevelt, R. & van Hemert, M. C. Photodissociation of water. I. electronic structure calculations for the excited states. *J. Chem. Phys.* **112**, 5777-5786 (2000).
19. Harich, S. A. et al., Photodissociation of H_2O at 121.6 nm: a state-to-state dynamical picture. *J. Chem. Phys.* **113**, 10073-10090 (2000).
20. Zahnle, K. J. & Walker, J. C. G. The evolution of solar ultraviolet luminosity. *Rev. Geophys. Space Phys.* **20**, 280-292 (1982).
21. Archer, L. E. et al., Room temperature photoabsorption cross section measurements

of CO_2 between 91,000 and 115,000 cm^{-1}. *J. Quant. Spectrosc. Radiat. Transf.* **117**, 88-92 (2013).
